# Mechanism(S) Involved in the Colon-Specific Expression of the Thiamine Pyrophosphate (Tpp) Transporter

**DOI:** 10.1371/journal.pone.0149255

**Published:** 2016-02-22

**Authors:** Svetlana M. Nabokina, Mel Brendan Ramos, Hamid M. Said

**Affiliations:** 1 Departments of Medicine, Physiology/Biophysics, University of California Irvine, Irvine, CA, 92697, United States of America; 2 Department of Veterans Affairs Medical Center, Long Beach, CA, 90822, United States of America; University of Florida, UNITED STATES

## Abstract

Microbiota of the large intestine synthesizes considerable amount of vitamin B1 (thiamine) in the form of thiamine pyrophosphate (TPP). We have recently demonstrated the existence of an efficient and specific carrier-mediated uptake process for TPP in human colonocytes, identified the TPP transporter (TPPT) involved (product of the *SLC44A4* gene), and shown that expression of TPPT along the gastrointestinal (GI) tract is restricted to the colon. Our aim in this study was to determine the molecular basis of the colon-specific expression of TPPT focusing on a possible epigenetic mechanism. Our results showed that the CpG island predicted in the *SLC44A4* promoter is non-methylated in the human colonic epithelial NCM460 cells, but is hyper-methylated in the human duodenal epithelial HuTu80 cells (as well as in the human retinal pigment epithelial ARPE19 cells). In the mouse (where TPPT expression in the GI tract is also restricted to the colon), the CpG island predicted in the *Slc44a4* promoter is non-methylated in both the jejunum and colon, thus arguing against possible contribution of DNA methylation in the colon-specific expression of TPPT. A role for histone modifications in the tissue-specific pattern of *Slc44a4* expression, however, was suggested by the findings that in mouse colon, histone H3 in the 5’-regulatory region of *Slc44a4* is tri-methylated at lysine 4 and acetylated at lysine 9, whereas the tri-methylation at lysine 27 modification was negligible. In contrast, in the mouse jejunum, histone H3 is hyper-trimethylated at lysine 27 (repressor mark). Similarly, possible involvement of miRNA(s) in the tissue-specific expression of TPPT was also suggested by the findings that the 3’-UTR of *SLC44A4* is targeted by specific miRNAs/RNA binding proteins in non-colonic, but not in colonic, epithelial cells. These studies show, for the first time, epigenetic mechanisms (histone modifications) play a role in determining the tissue-specific pattern of expression of TPPT in the GI tract.

## Introduction

The water-soluble vitamin thiamine (vitamin B_1_) is essential for cellular metabolism mainly through of its role as a co-factor (in the form of thiamin pyrophosphate/diphosphate) for multiple enzymes that catalyze oxidative energy metabolism, and of reducing cellular oxidative stress [1–5]. Thus, it is not surprising that a deficiency of this essential micronutrient (which occurs in conditions like chronic alcoholism, diabetes mellitus, and at other risk populations, such as in the elderly, patients after chronic liver failure, etc.) leads to serious clinical abnormalities that include neurological and cardiovascular disorders [1, 6–10].

Humans and other mammals cannot synthesize thiamine endogenously; rather they obtain this micronutrient from exogenous sources via intestinal absorption. Two sources of thiamine are available to the gut: a dietary source and a microbiota source. Dietary thiamine is absorbed in the small intestine (after conversion of the phosphorylated forms of the vitamin to free thiamine by the abundant small intestinal phosphatases) via a specific carrier-mediated process that involves the thiamine transporter-1 and -2 systems (reviewed in [7–9, 11]). A considerable amount of the thiamine generated by the gut microbiota exists in the pyrophosphate (TPP) form [12]. Recent studies from our laboratory have shown that human colonocytes possess an efficient and specific carrier-mediated process for uptake of the microbiota generated TPP [13]. We have also established the molecular identity of the colonic TPP transporter (TPPT) as the product of the *SLC44A4* gene [14], and have further cloned and characterized its 5’-regulatory region and showed an important role for the ETS/ELF3 (E74-Like Factor 3 (Ets Domain Transcription Factor, Epithelial-Specific)) and CREB-1 (cAMP-responsive element-binding protein-1) transcriptional factors in regulating the basal activity of the *SLC44A4* promoter [15]. While examining the pattern of expression of the TPPT along the human and mouse GI tract, we found that expression of this system is mainly restricted to the colon with negligible expression in other parts of the GI tract [14]. Little is known about the molecular mechanism(s) involved in determining this colon-specific pattern of expression of the TPPT system. Our aim was to investigate this issue focusing on a possible role of epigenetic mechanisms and microRNAs, since these two mechanisms appear to play an important role in determining tissue-specific expression of other transporters [16–23]. Here, we determined the DNA methylation and histone H3 modification profiles in the promoter regions of the human and mouse TPP transporter genes, and estimated the possible contribution of miRNAs towards the regulation of TPPT expression in the intestine. Our results showed that the high colon-specific expression of TPPT and the absence/negligible expression of this transporter in other parts of GI tract are associated with certain histone H3 modifications in its promoter region, while the gene regulation through promoter methylation appears to play no role. In addition, the results provide clear evidence that TPPT expression is not regulated by miRNAs in the colon, but has a potential to be down-regulated by miRNAs/RNA binding proteins in a cell-specific manner.

## Materials and Methods

### Materials

Human-derived colonic epithelial NCM460 cells were from INCELL (San Antonio, TX). Human-derived intestinal epithelial Caco2 and HuTu80 cells, retinal pigment epithelial ARPE19 cells, and lung epithelial A549 cells were purchased from American Type Culture Collection, ATCC (Manassas, VA). pmirGLO Dual Luciferase miRNA target expression vector was from Promega (Madison, WI). The antibodies for histone H3, H3K4me3, H3K9Ac, H3K27me3 and normal rabbit IgG were purchased from Millipore (Billerica, MA).

### Cell Culture, Transfection, and Reporter Gene Assay

Caco2, HuTu80, and ARPE19 cells were grown in Dulbecco's modified Eagle's medium (DMEM) supplemented with 10% (vol/vol) FBS, penicillin (100,000 U/l), and streptomycin (10 mg/l). NCM460 cells were maintained in Ham's F-12 culture medium supplemented with 20% (vol/vol) FBS and antibiotics. A549 cells were maintained in F-12K medium (Kaighn’s modification of Ham's F-12 medium) supplemented with 10% (vol/vol) FBS and antibiotics. For regular maintenance, cells were grown in 75-cm^2^ plastic flasks at 37°C in a 5% CO_2_-95% air atmosphere with media changes every 2–3 days.

For 5-azacytidine treatment and transient transfection, cells were plated at a density of ~2 × 10^5^ cells/well onto 12-well tissue culture plates (Corning, NY). Cells were treated with 5-azacytidine (0, 1, and 10 μM) for 48 hrs followed by RNA isolation. For transient transfection, ~80–90% confluence cells were transfected with 2 μg of plasmid DNA (pmirGLO-3’UTR-SLC44A4 or pmirGLO vector) with the Lipofectamine 2000 (Invitrogen, Carlsbad, CA) according to the manufacturer’s instructions. *Renilla*-normalized firefly luciferase activity was measured 48 hours after transfection by using the Dual Luciferase Assay system (Promega). Data are presented as means ± SE of at least three independent experiments. The Student's *t*-test was used for statistical analysis, and *P* < 0.05 was considered statistically significant.

### Quantitative Real-Time PCR Analysis

Total RNA was isolated from cells treated with 5-azacytidine and subjected to reverse transcription using the iScript cDNA synthesis kit (Bio-Rad, Hercules, CA). The hTPPT mRNA expression level was quantified in a CFX96 real-time PCR system (Bio-Rad) using iQ SYBR Green Supermix (Bio-Rad) and primers specific for hTPPT (forward: 5′-TGCTGATGCTCATCTTCCTGCG-3′ and reverse: 5′-GGACAAAGGTGACCAGTGGGTA-3′), and β-actin (forward: 5′-AGCCAGACCGTCTCCTTGTA-3′ and reverse: 5′-TAGAGAGGGCCCACCACAC-3′). Conditions were as previously described [24]. Data normalized to β-actin were quantified using a relative relationship method supplied by the iCycler manufacturer (Bio-Rad). The Student's *t*-test was used for statistical analysis, and *P* < 0.05 was considered statistically significant.

### Cloning of the 3’-Untranslated Region (3’-UTR) of the SLC44A4 Gene

A 431-bp region of the SLC44A4 3’-UTR was amplified by PCR from cDNA derived from NCM460 cells. The PCR cloning primers were as follows: forward 5’–CCGAGCTCCAGCTCCGGCCCTGATCCAGGACTGC– 3’ and reverse 5’–CCGTCGACACTGAGTTAACAAAATATCTTTAATA– 3’. The generated PCR product was purified, digested with *SacI* and *SalI* (these sites were designed into the primers), and inserted into multiple cloning sites of a pmirGLO miRNA target expression vector downstream of the luciferase reporter gene. The DNA sequence was verified by the Laragen Sequencing Facility.

### DNA Methylation Pattern

Various human-derived cell lines and intestinal mucosa from jejunum and proximal colon of mice were used for genomic DNA isolation. Animal studies were approved by the Institutional Animal Care Use Committee (IACUC) of the Long Beach VA Medical Center. Genomic DNA was isolated using Wizard Genomic DNA purification kit (Promega) according to the manufacturer’s instructions and then subjected to bisulfite reaction utilizing EpiTect Bisulfite kit (Qiagen). By the bisulfite reaction unmethylated cytosine residues are changed to uracils in the resultant product. The CpG islands identified in the promoter region of human *SLC44A4* and mouse *Slc44a4* genes by *in silico* CpG island prediction/primer design Methprimer algorithm [25] were then amplified by nested PCR from bisulfite-modified genomic DNA. The primers were as follows: human CpG island (forward primer 5’-TTAGGTTTTTAGGGTTTTAATTATTT-3’ and reverse primer 5’-TCTCCTTAATTCCTCTCCCTAAAAC-3’) and mouse CpG island (forward primer 5'- GGTATTTTTGGGTTGGTTAAGAGTT -3’ and reverse primer 5'- TACTTTCTCCCCATAACTCAATCTC-3’). The generated PCR products were inserted into the pGEM-T Easy vector (Promega). Randomly selected clones (at least 10 and 7 clones for individual human and mouse CpG island sample, respectively) were sequenced by the Laragen Sequencing Facility. The DNA methylation pattern of the CpG islands was determined using the QUMA methylation analysis tool [26]. Data presented are from studies performed on two or more separate occasions with similar results.

### ChIP Assay

ChIP was performed with the SimpleChIP^™^ Enzymatic Chromatin IP Kit (Cell Signaling, Danvers, MA) according to manufacturer’s protocol. Briefly, freshly scraped intestinal mucosa from mouse jejunum and proximal colon (~20 mg per each immunoprecipitation) was treated with 1% formaldehyde (final concentration) for 10 min (to crosslink proteins to DNA), followed by disaggregating of the tissue and nuclei isolation from the resultant cell suspension as per the instructions of the kit. The chromatin was digested with the Micrococcal Nuclease, followed by sonication, to shear DNA to fragments of ~150–900 bp in length (the DNA fragment size was verified by 1% agarose gel electrophoresis after DNA purification). Chromatin sample (~ 6 μg per immunoprecipitation) was incubated with 2 μg of H3, H3K4me3, H3K9Ac, H3K27me3 antibody or normal rabbit IgG overnight at 4°C, followed by precipitation of immunocomplex on protein G agarose beads for 2 hrs at 4°C. Immunoprecipitates were eluted and all samples (including 2% input sample) were subjected to reversal of protein/DNA crosslinks and DNA purification as per manufacturer’s protocol. The resultant DNA samples were then subjected to quantitative real-time PCR using primers for amplification of the 5’-regulatory region of mouse *Slc44a4* gene: 5’-TAGGTCTCCGTGGCTCCAAT-3’ (forward) and 5’-CCGTGAGCCTCGTTCTCATT -3’ (reverse). Conditions were as previously described [24]. The qPCR results were analyzed using the Percent Input Method, where the amount of immunoprecipitated DNA in each sample is represented as signal relative to the total amount of input chromatin (data are given in % relative to a qPCR on input DNA). The data are expressed as the means ± S.E. of triplicate experiments and presented together with the background signal (normal rabbit IgG) to verify the immunoprecipitation specificity.

## Results

### DNA Methylation Profiles in the Promoter Regions of the Human and Mouse TPP Transporter Genes

**Fig 1:**
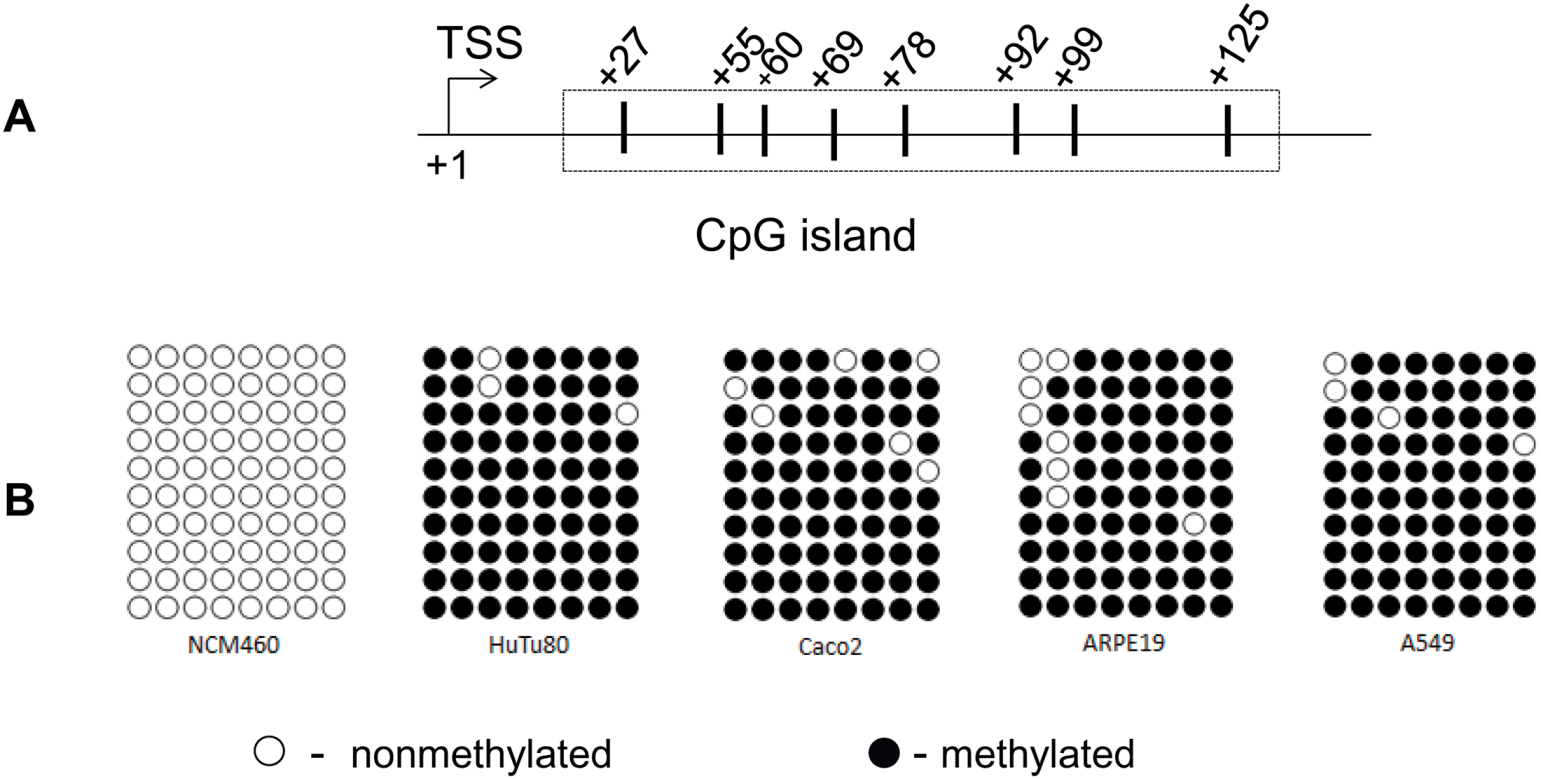
DNA methylation profiles of the SLC44A4 promoter in different human epithelial cell lines. **(A)** Schematic representation depicting the predicted CpG island in the promoter region of the *SLC44A4* gene by the Methprimer algorithm (25). The positions of the CpG dinucleotides are shown with vertical lines; numbers indicate the location of cytosine residues relative to the transcription start site, TSS (+1) (15). **(B)** Methylation status of the CpG dinucleotides. Genomic DNA was isolated from different human epithelial cell lines and the methylation profiles in the CpG dinucleotides clustered inside the targeted island were analyzed by bisulfite sequencing. The number of symbols indicates the number of sequenced PCR products. ○ –nonmethylated; ● –methylated.

An *in silico* CpG island prediction/primer design Methprimer algorithm [25] was used to predict the CpG islands in the promoter region of the *SLC44A4* gene. Experimentally verified 1,022 bp *SLC44A4* promoter [15] was subjected to prediction algorithm analysis using the default parameters. The analysis identified a single CpG island that was defined in accordance with the classical sequence parameters of the CpG islands [27, 28]. The identified CpG island (Fig 1A) has an elevated G+C content (> 70%), a CpG frequency (observed/expected, [o/e]) of > 0.6, and was located in close proximity to the TSS.

We studied the DNA methylation status of this CpG island of *SLC44A4* gene in human epithelial cells. Genomic DNA was isolated from intestinal epithelial NCM460, Caco2 and HuTu80 cells, retinal pigment epithelial ARPE19 cells, and lung epithelial A549 cells and methylation profiles in the CpG dinucleotides clustered in the targeted island were analyzed by bisulfite sequencing. The results of bisulfite sequencing analysis (Fig 1B) showed that the CpG island associated with the promoter region of *SLC44A4* gene is nonmethylated in colonic NCM460 cells, but considerably hypermethylated in all other examined cells including intestinal epithelial Caco2 and HuTu80. This DNA methylation profile is consistent with the colon-specific expression of SLC44A4 transporter and the absence/negligible expression of this transporter in non-colonic cells [14].

**Fig 2:**
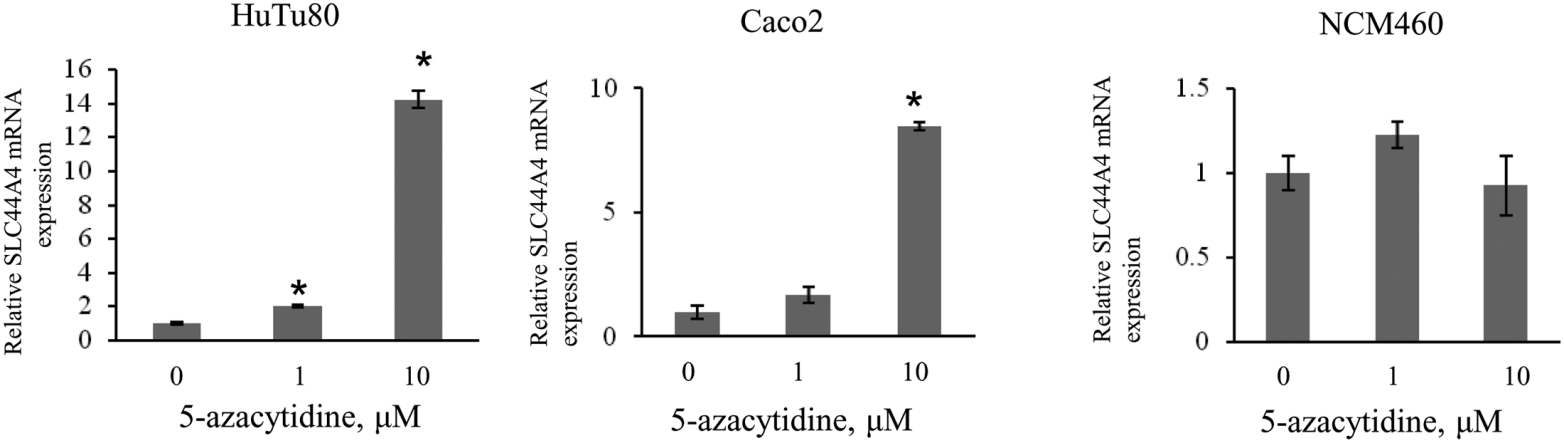
Effect of 5-azacytidine on the SLC44A4 mRNA expression. Cells were treated with 5-azacytidine (0, 1, and 10 μM) for 48 hrs followed by RNA isolation and quantitative RT-PCR analysis. Results, normalized relative to β-actin, are presented as fold increase compared to untreated control (set as 1). Data are means ± SE from three independent experiments. *P < 0.05.

To further estimate the possible role of DNA methylation in suppressing the *SLC44A4* gene activity in non-colonic intestinal cells, we studied the effect of DNA demethylation on the *SLC44A4* mRNA expression. Cultured non-colonic intestinal Caco2 and HuTu80 cells (both non-expressing SLC44A4) were treated with the DNA methyl transferases inhibitor 5’-azacytidine, followed by quantitative RT-PCR analysis. The results (Fig 2) showed that treatment with demethylating agent resulted in a significant (P < 0.05) induction of the *SLC44A4* mRNA in a dose-dependent manner. In contrast, there was no further induction in *SLC44A4* expression in NCM460 cells after treatment with 5’-azacytidine. These data suggest that there is a correlation between the *SLC44A4* gene activity and the extent of methylation of its promoter in human intestinal epithelial cell lines.

To verify the physiological relevance of these data obtained *in vitro* with the human-derived cell lines, we studied the DNA methylation pattern of the 5’-regulatory region/promoter region of the mouse *Slc44a4* gene in native mouse intestine. These studies were performed utilizing the intestinal mucosa from jejunum (negligible *Slc44a4* expression) and proximal colon (high *Slc44a4* expression) of mice [14].

**Fig 3:**
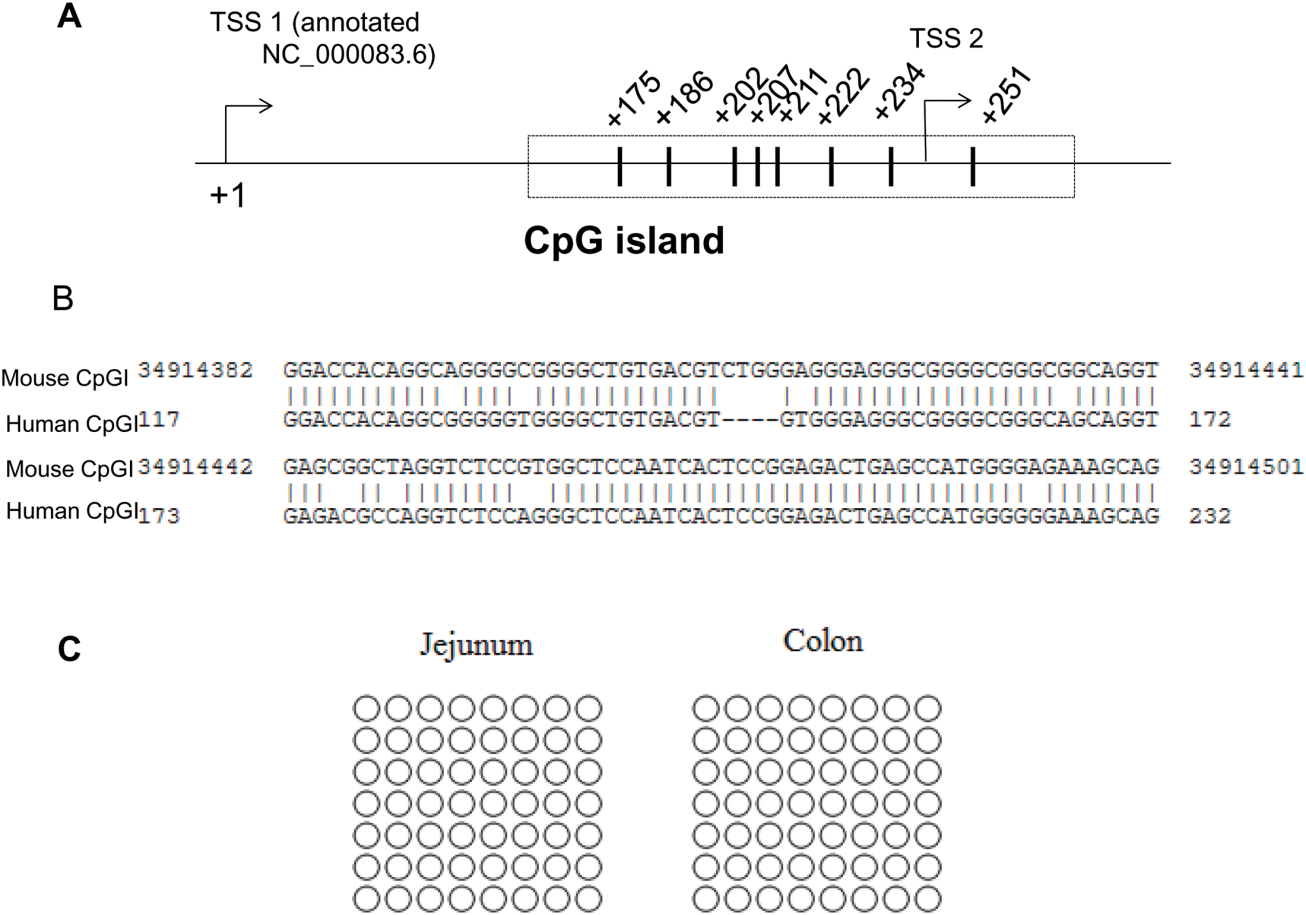
DNA methylation pattern of the 5’-regulatory /promoter region of the mouse Slc44a4 gene in native mouse intestine. **(A)** A schematic representation of the CpG island, predicted in the 5’-regulatory region of the mouse *Slc44a4* gene (GeneBank accession no. NC_000083.6) by Methprimer algorithm (25). Two TSS positions are shown; +1 refers the distal site. CpG positions are indicated with numbers relative to TSS (+1). **(B)** The BLASTN comparison of the mouse *Slc44a4* 5’-regulatory region with those of human *SLC44A4* (using the 266-bp minimal *SLC44A4* promoter region (15) with the first nucleotide of this region numbered as 1). A significant sequence similarity was found in their predicted CpG islands (shown) with no similarity outside the islands. **(C)** Methylation status of CpG dinucleotides. Genomic DNA was isolated from intestinal mucosa from jejunum and proximal colon of mice; methylation profiles of the CpG dinucleotides located in the CpG island were determined by bisulfite sequencing. The number of symbols indicates the number of sequenced PCR products. ○ –nonmethylated.

First, we examined whether the 5’-regulatory/promoter region of the mouse *Slc44a4* gene (region between 34914239 and 34930436 nt; GeneBank accession no. NC_000083.6) contains the CpG islands, as it was found to be a case for human *SLC44A4* gene. Our analysis using the CpG island prediction/primer design Methprimer algorithm [25] led to the identification of a single CpG island located between -85 nt and +32 nt (relative to proximal TSS set as +1) (Fig 3A). This predicted CpG island localized to the annotated TSSs and revealed the sequence parameters of the CpG islands: GC content of > 60% and a ratio > 0.6 of observed number of CpG dinucleotides to the expected number. To investigate whether the 5’-regulatory/promoter regions of the human and mouse TPP transporter genes have homology, we compared their nucleotide sequences using Basic Local Alignment Search Tool (BLAST). The BLASTN comparison reveals a high degree of similarity (*i*.*e*. 84% of identity) between the human and mouse promoters in their regions that were identified as the CpG islands (Fig 3B). Outside the predicted CpG islands, no significant sequence similarity was found.

We studied the methylation status of the CpG island associated with the *Slc44a4* 5’- regulatory region in mouse intestinal segments. Sequencing analysis using the bisulfite converted genomic DNA prepared from freshly scrapped jejunal and proximal colonic mucosa revealed that the CpG island of the *Slc44a4* gene is nonmethylated in both jejunum and colon (Fig 3C). This discrepancy in the methylation status of the CpG island observed between native small intestine and cultured cells (used as an *in vitro* model for duodenum/small intestine in our experiments) could be due to the transformed nature of the cell lines used in these studies [29, 30]. Our data obtained with native mouse intestine indicate that the high colon-specific expression of TPPT and absence/negligible expression of this transporter in other parts of GI tract cannot be explained through gene silencing by DNA methylation. This led us to aim at investigating possible involvement of epigenetic mechanisms other then DNA methylation in the tissue-specific pattern of TPPT expression.

### Histone H3 Modification Profiles in the Promoter Region of the Mouse TPP Transporter Gene

**Fig 4:**
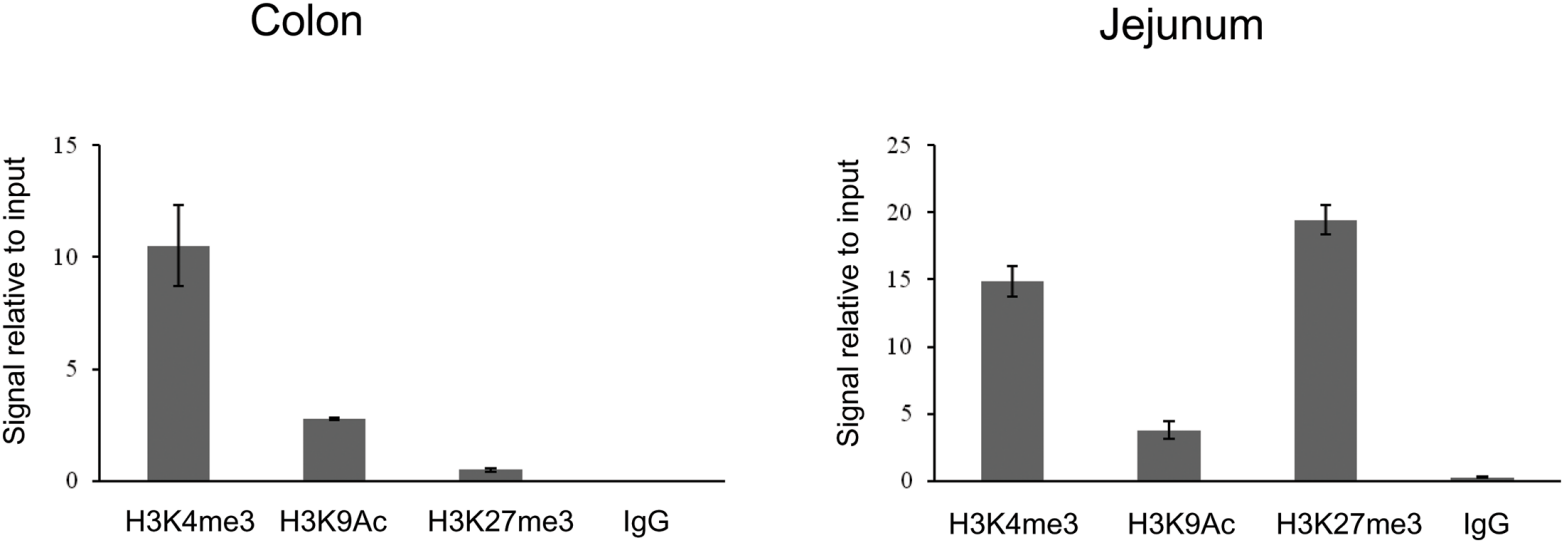
Analysis of histone H3 modifications in the colon and jejunum. ChIP assays using freshly scrapped mouse mucosa from jejunum and proximal colon and antibodies specific for modified forms of histone H3, followed by quantitative real-time PCR, were performed as described under *Materials and Methods*. The qPCR results were analyzed using the Percent Input Method and are presented in % relative to a qPCR on input DNA. The data are expressed as the mean ± S.E. of triplicate experiments. The background signal (normal rabbit IgG) was included to verify the immunoprecipitation specificity.

It is known that CpG islands acquire various chromatin states each with varying propensities for gene expression [27, 28, 31, 32]. Thus, we studied the chromatin state of the CpG island associated with the 5’regulatory region of *Slc44a4* gene in mouse jejunum and colon. We examined histone H3 modifications that are broadly used in epigenetic studies and are characteristic for active chromatin architecture, i.e. trimethylation at the lysine 4 (H3K4me3) and acetylation at lysine 9 (H3K9Ac), as well as for repressed chromatin status, i. e. trimethylation at lysine 27 (H3K27me3). Furthermore, the H3K27 trimethylation was used because it has been reported to serve as an epigenetic mark of transcription repression in a cell-specific manner [33]. We performed ChIP assays using freshly scrapped mouse mucosa from jejunum and proximal colon and antibodies specific for modified forms of histone H3, followed by quantitative PCR. The results of qPCR analysis (Fig 4) showed that in the mouse colon, histone H3 in the 5’regulatory region of *Slc44a4* is trimethylated at lysine 4 and acetylated at lysine 9, whereas the tri-methylation at lysine 27 modification was found to be negligible. In contrast, in the mouse jejunum, histone H3 is hypertrimethylated at lysine 27 (repressor mark). These data on histone H3 modification profiles are consistent with the tissue-specific expression pattern of the *Slc44a4* gene and indicate that its tissue-specific transition to the active or the repressed state appears to occur through certain histone modifications.

### 3’-UTR of SLC44A4 Is Targeted by Specific miRNAs/RNA Binding Proteins in Non-Colonic, but Not in Colonic, Epithelial Cells

**Fig 5:**
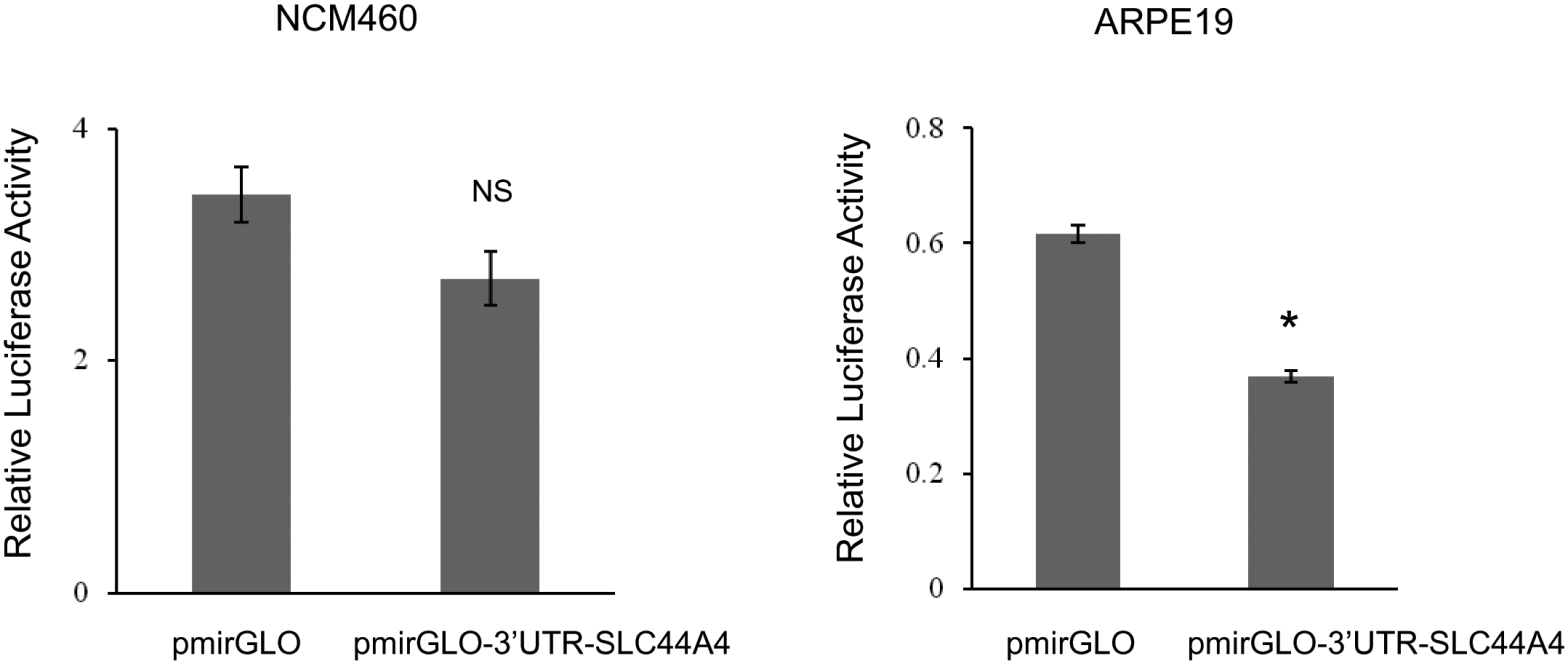
Effect of the 3’-UTR of SLC44A4 on luciferase reporter activity. Constructs, pmirGLO-3’UTR-SLC44A4 or pmirGLO vector (control), were transiently expressed for 48 hours in NCM460 and ARPE19 cells for luciferase assays. Data are reported as relative firefly luciferase activity normalized to *Renilla* luciferase activity and represent means ± SE of at least 3 independent experiments, each performed in triplicate. *P < 0.05. NS, not significant.

Nothing is known about potential involvement of miRNAs in regulation of TPPT expression in any cells/tissues. Here we addressed the potential role of miRNAs in the tissue-specific regulation of *SLC44A4*. We first examined whether the 432-bp 3’-UTR of the *SLC44A4* transcript contains the putative binding sites for miRNAs. To improve the specificity of our *in silico* identification of putative binding sites for miRNAs, we used multiple miRNA target prediction programs (miRDB, TargetScanS, miRanda, DIANA microT-CDS) since no one individual program was superior to the rest. Based on context scores and context score percentile as well as the outcome from multiple program prediction analysis, certain miRNAs, such as hsa-miR-4638-5p, hsa-miR-342-5p, hsa-miR-1976, hsa-miR-125a-3p and others were predicted to efficiently target the SLC44A4 3’-UTR. These identified miRNAs are not well characterized, and there is no data on their expression in the GI tract and thus, their potential to serve in down regulation of *SLC44A4* gene expression in intestine. As a first step into gaining knowledge on the SLC44A4 regulation by miRNAs, a 432-bp of the SLC44A4 3’-UTR was amplified from total RNA of NCM460 cells and cloned into a pmirGLO expression vector. The colonic NCM460 and non-colonic ARPE19 cells were transiently transfected with the generated pmirGLO-3’UTR-SLC44A4 construct followed by Luciferase measurements. As shown in Fig 5, no significant decrease of relative luciferase reporter activity was observed in NCM460 cells transfected with SLC44A4 3’-UTR compared to control (cells transfected with the empty pmirGLO vector). In contrast, in non-colonic ARPE19 cells a significant decrease in luciferase activity (~40% decrease) was observed (Fig 5). These data indicate that although the 3’-UTR of SLC44A4 can be targeted by miRNAs/RNA binding proteins (as it occurs in ARPE19 cells), the set of miRNAs presented in colonic cells do not appear to bind to the 3’-UTR of SLC44A4, and do not affect the SLC44A4 mRNA level there.

## Discussion

Our aim in this study was to establish the molecular basis of the colon-specific expression of the TPPT in the intestinal tract. Previously, we identified two transcription factors, i.e. ELF3 and CREB-1 that play key roles in the regulation of the *SLC44A4* basal promoter activity in human colonic epithelial cells [15]. Based on our findings of high expression of these transcription factors in colonic epithelial (NCM460) cells compared to non-colonic epithelial (ARPE19) cells, we believed that ELF3 and CREB-1 may contribute to the tissue-specific pattern of SLC44A4 expression. However, regarding the GI tract, the high *SLC44A4* gene expression in the colon and its negligible expression in the small intestine could not be explained based on differential expression of the above-described nuclear factors. Indeed, the CREB-1 is not expressed exclusively in colon, but is also expressed in other tissues (including small intestine) [34], and the ELF3 factor has a unique epithelium-restricted pattern of expression with particularly strong expression in both the small intestine and colon [35, 36]. Thus, factor(s)/system(s) other than the transcription factor network contribute toward establishing the colon-specific *SLC44A4* gene expression pattern throughout the intestinal tract. In the current study we considered a possible role of epigenetic mechanisms (DNA methylation and histone modifications), as well as miRNAs in the establishment of the tissue-specific pattern of TPPT expression.

Methylation of CpG dinucleotides in the 5’-regulatory/promoter region is known to correlate with the condensed structure of chromatin and down-regulation/silencing of the downstream gene expression. The unique DNA sequences with high CpG densities (CpG islands) overlap the promoter regions of the majority of human genes with the large portion of those representing tissue-specific expression [28, 31, 32]. CpG islands generally lack DNA methylation, and thus, are characterized by a transcriptionally permissive chromatin state. But a small percentage of CpG islands become methylated during normal development [37]. Furthermore, recent studies have reported the differential CpG island methylation in somatic cells that contribute into a tissue-specific pattern of CpG island methylation [37–39]. Thus, we checked if this is the case for the TPPT gene. Using the *in silico* CpG island prediction/primer design Methprimer algorithm [25], we found that the promoter region of the human *SLC44A4* gene contains a single CpG island that was defined in accordance with the classical sequence parameters of the CpG islands. We studied the DNA methylation status of this identified CpG island in human epithelial cells by bisulfite sequencing and found that the CpG island is nonmethylated exclusively in colonic NCM460 cells; all other examined cells including intestinal epithelial Caco2 and HuTu80 demonstrated hypermethylation in this CpG island. We also found that there is an inverse correlation between the *SLC44A4* gene activity and extent of methylation of its promoter in human intestinal epithelial cell lines. Indeed, treatment with the DNA methyl transferase inhibitor 5’-azacytidine significantly increased the *SLC44A4* mRNA level in non-expressing cells. The observed DNA methylation profile is consistent with the previously reported tissue-specific expression of SLC44A4 transporter [14]. However, these *in vitro* data on DNA methylation status of the CpG island associated with the *SLC44A4* promoter obtained with the human-derived cell lines, were found to have no relevance to the *in vivo* situation with native jejunal and colonic mouse tissues. Indeed, the CpG island identified in the 5’-regulatory region/promoter region of the mouse *Slc44a4* gene (which share 84% sequence identity with the CpG island of the human *SLC44A4* gene) was found to be nonmethylated in both the jejunum and the colon. Thus, the high *Slc44a4* expression in colon and negligible *Slc44a4* expression in jejunum [14] cannot be explained via the differential DNA methylation status of the transporter gene; thus, a physiological role of DNA methylation as the possible epigenetic mechanism can be excluded. The observed discrepancy in the DNA methylation data obtained *in vitro* and *in vivo* could be due to the transformed nature of all cell lines (except NCM460 cells) used in these studies. The reasons that lead to the *SLC44A4* promoter inactivation by DNA methylation *in vitro* are unclear. Since the aberrant promoter hypermethylation in malignancy is thought to contribute to carcinogenesis by inactivating tumor-suppressor genes [29, 30], there is a possibility that the hTPPT functions as a tumor suppressor. Another possibility is that the examined cells (except colonic cells) do not need a TPP transporter for their functionality resulting in hypermethylation of the promoter and gene silencing. Further studies are needed to address these possibilities.

Modifications of the N-terminal tails of histones are generally recognized epigenetic marks that play important roles in transcriptional regulation of gene expression. Certain histone modifications in the promoter regions of genes, in particular around the TSS and in the promoter-associated CpG islands, are known to correlate strongly with the transcriptional activity [28, 31–33]. Furthermore, recent studies showed that the CpG islands regulate transcriptional potential of the associated gene in many physiological processes, including differentiation and tissue-specificity establishment, and, byacquiring various chromatin states, determine transition of the associated gene to the active or repressed state during development [28, 31–33]. In this study, we performed the comparative analysis (colon versus jejunum) histone H3 modification profiles of the CpG island associated with the 5’regulatory region of mouse *Slc44a4* gene and found different levels of characteristic histone modifications in mouse colon and jejunum. The analysis in colon showed high levels of histone H3K4me3 and H3K9Ac modifications, which are characteristic of active chromatin, and very low/barely detectable levels of histone H3 modification, which is a distinctive feature of inactive chromatin, H3K27me3. On the other hand, in jejunum the level of H3K27me3 modification was found to be highly elevated. Interestingly, in jejunum we also observed a considerable level of H3K4me3 modification (euchromatin marker). This was expected, because H3K4me3 is a signature mark of CpG island–associated promoters, and this type of histone H3 modification is often present even when the gene is transcriptionally inactive [40, 41]. Our data on histone H3 modification profiles are in agreement with the tissue-specific expression pattern of the *Slc44a4* gene, confirming its active transcription in colon and repressed transcription in jejunum. Also, these data suggest that certain histone H3 modifications appear to contribute into transitioning the nonmethylated, and thus transcriptionally permissive CpG island of *Slc44a4* gene, to the active or the repressed state in a tissue-specific manner in the intestinal tract.

MicroRNAs are small non-coding RNAs of 19–24 nucleotides which inhibiting the translation or mediating degradation of their target protein-coding mRNAs, and thus negatively regulate gene expression at the posttranscriptional level [42, 43]. To date, about half of mammalian protein coding-genes have been estimated to be regulated by miRNAs. Specific miRNAs are expressed in the intestinal epithelial cells (including colonocytes) where they regulate expression of genes implicated in the vital cellular processes/functions such as intestinal epithelial cells differentiation, barrier functions, transport functions, apoptosis, and cell-to-cell communication during colonic inflammation [44–47]. Nothing, however, is known about possible involvement of miRNAs in regulation of TPPT expression. In this study, we obtained evidence that the 3’-untranslated region (3’-UTR) of *SLC44A4* gene contains binding sites for miRNAs/RNA binding proteins, and *in silico* identified the putative miRNAs predicted to target the 3’-UTR of *SLC44A4*. We also obtained evidence suggesting that miRNAs/RNA binding proteins targeting the SLC44A4-3’-UTR is cell-specific. This is based on the observations that the 3’-UTR of SLC44A4 can be targeted by miRNAs/RNA binding proteins, as observed in retinal epithelial ARPE19 cells (SLC44A4 non-expressing cells), leading to down-regulation of gene expression, on the other hand, this does not appear to happen in colonic epithelial cells as indicated by the findings with the NCM460 cells showing that the set of miRNAs that exist in these cells do not bind to the 3’-UTR of SLC44A4 and do not affect/downregulate SLC44A4 mRNA expression (i. e., data are in agreement with high expression in colon).

In summary, results of these investigations show, for the first time, that epigenetic mechanisms (histone modifications) play a role in determining the tissue-specific pattern of expression of the TPPT along the GI tract.
