# Correction: Mechanism(S) Involved in the Colon-Specific Expression of the Thiamine Pyrophosphate (Tpp) Transporter

**DOI:** 10.1371/journal.pone.0186550

**Published:** 2017-10-11

**Authors:** Svetlana M. Nabokina, Mel Brendan Ramos, Hamid M. Said

The following information is missing from the Funding section: This study was supported by NIH grant AA 18071 to HMS.

## References

[1] Nabokina SM, Ramos MB, Said HM (2016) Mechanism(S) Involved in the Colon-Specific Expression of the Thiamine Pyrophosphate (Tpp) Transporter. PLoS ONE 11(2): e0149255 https://doi.org/10.1371/journal.pone.0149255 26901654 10.1371/journal.pone.0149255 PMC4764741

